# Apolipoprotein-A is a potential prognostic biomarker for severe aplastic anemia patients treated with ATG-based immunosuppressive therapy: a single-center retrospective study

**DOI:** 10.1186/s12944-022-01703-0

**Published:** 2022-10-04

**Authors:** Qi Liu, Huijie Dong, Yuzhu Li, Yingying Shen, Yilei Hong, Ying Chen, Shan Liu, Xiaolian Wu, Wenbin Liu, Huijin Hu, Yuechao Zhao, Shenyun Lin, Yiping Shen, Yuhong Zhou, Baodong Ye, Dijiong Wu

**Affiliations:** 1 The First School of Clinical Medicine, Zhejiang Chinese Medical University, Hangzhou, Zhejiang China; 2 Department of Respiratory Medicine, Haining Traditional Chinese Medical Hospital of Zhejiang Province, Haining, Zhejiang China; 3 Department of Hematology, The First Affiliated Hospital of Zhejiang Chinese Medical University, Hangzhou, 310005) Zhejiang People’s Republic of China; 4 Department of Clinical Evaluation Center, the First Affiliated Hospital of Zhejiang Chinese Medical University, Hangzhou, Zhejiang, China

**Keywords:** Blood lipid, Apolipoprotein-A, Aplastic anemia, T-cell subset, Immune globulin

## Abstract

**Background:**

Anti-thymoglobulin (ATG)-based immunosuppressive treatment (IST) is the standard first-line management for patients with severe AA/very severe AA (SAA/VSAA) and is not suitable for allogeneic stem cell transplantation. The response predictor was not fully investigated.

**Objective:**

The present study attempted to explore other characteristics, such as serum lipid changes, during ATG-based IST and analyzed their significance in predicting IST response and survival.

**Methods:**

A total of 61 newly diagnosed SAA/VSAA patients who received ATG-based IST were enrolled from January 2011 to June 2019. The blood lipid levels, immunoglobulins, and peripheral T lymphocytes were retrospectively collected, and their correlations with IST response, estimated 8.5-year overall survival (OS) and event-free survival (EFS) were analyzed.

**Results:**

The overall response (OR)/complete remission (CR) at 3, 6, and 9 months was 24.6%/6.6%, 52.5%/14.8%, and 65.6%/23.0%, respectively. Based on the 9-month response effect, patients were divided into IST-response (IST-R) and IST-nonresponse (IST-NR) groups. The subgroup baseline characteristics showed that the disease severity grade, absolute neutrophil granulocyte count (ANC), total cholesterol (TC), low-density lipoprotein cholesterol (LDL-C), high-density lipoprotein cholesterol (HDL-C), and apolipoprotein-A (Apo-A) differed between the IST-R and IST-NR groups. Patients with lower Apo-A (< 1.205 g/L) level pretreatment had a better event-free survival (EFS), and a moderate negative correlation was established between the pretreatment Apo-A and 9-month response (*P* = 0.004). In addition, the T-cell subset and immunoglobulin analyses showed that the responsive patients had a low serum IgA level, which decreased further after therapy. Additionally, a moderate negative correlation was established between the 3-month IgA and 9-month response (*P* = 0.006).

**Conclusion:**

Serum Apo-A is a prognostic biomarker for newly diagnosed < 60-year-old SAA/VSAA patients who received ATG-based IST (registered at *chictr.org.cn as # ChiCTR2100052979*).

**Supplementary Information:**

The online version contains supplementary material available at 10.1186/s12944-022-01703-0.

## Introduction

Aplastic anemia (AA) is a hematopoietic failure disease characterized by pancytopenia with a risk of hemorrhage and infection. AA is further classified into severe AA (SAA) and nonsevere AA (NSAA) based on the severity of the disease. SAA patients with an absolute neutrophil count (ANC) < 0.2 × 10^9^ meet the criterion of very severe AA (VSAA) [1]. The current standard first-line management for SAA/VSAA is anti-thymoglobulin (ATG)-based immunosuppressive treatment (IST) or hematopoietic stem cell transplantation (HSCT). Patients aged 35–50 years should be assessed fully for comorbidities before considering transplantation. Nonetheless, ATG-based IST is a feasible option for patients without sibling-matched donors (MSDs) [2].

The efficiency of ATG-based IST was 52–88% [3, 4], and 1/5–1/3 patients did not achieve a response. Several studies have identified the influencing factors on response and survival, including patient age, disease severity, absolute reticulocyte (Ret) count, and the interval between diagnosis and treatment [3, 5, 6]. All these predictors emphasized the importance of residual hematopoietic function for the IST response. However, patients still exhibit poor response even if they are supposed to respond well to ATG-based IST. Based on the mechanism of the disease, cytotoxic T lymphocyte-mediated hematopoietic stem cell (HSC) exhaustion is the major pathogenesis [7]. However, other factors, including cytogenetic background differences and hematopoietic microenvironment impairment, may contribute to the prognosis [8–10]. For the former factor, HSCT may be beneficial, while for the hematopoietic microenvironment, many aspects, such as iron overload/lipid-induced oxidative stress and adipogenic/osteogenic differentiation balance of mesenchymal stem cells (MSCs) [11, 12], need to be investigated. An increasing number of scholars have realized that lipid metabolism is essential in regulating HSC function, particularly in maintaining HSC asymmetric division for self-renewal and differentiation capacity by fatty acid oxidation (FAD) [13]. However, the role of lipid metabolism in diseases with abnormal hematopoietic stem cell function has yet to be elaborated. Hence, the purpose of this study was to explore the serum lipid changes during ATG-based IST and identify putative indicators that may predict prognosis, thereby implying that they are potential therapeutic targets.

## Methods

### Study subjects

All consecutive patients with a diagnosis of SAA or VSAA who received ATG-based immunosuppressive therapy from January 2011 to June 2019 participated in this retrospective observational cohort study. The inclusion criteria were as follows: (1) age between 10 and 60 years old; (2) newly diagnosed within 3 months after the first complaint; (3) diagnosis of SAA or VSAA that met Camitta criteria [1]; and (4) received ATG-based IST. The exclusion criteria were as follows: (1) disease progressed from non-SAA; (2) secondary pancytopenia developed from other hematological or immunological diseases; and (3) early death (died within 1 month after ATG-based IST) led to an inability to evaluate the treatment efficiency. The trial was registered at *chictr.org.cn* as # *ChiCTR2100052979*.

### Laboratory investigations

Blood samples were collected from the cubital vein of patients under the condition of overnight fasting for ≥ 12 h. Routine blood tests were performed every three days (closer if necessary) on a hematology analyzer (Beckman, DxH800). Triglycerides (TGs), total cholesterol (TC), low-density lipoprotein-cholesterol (LDL-C), high-density lipoprotein-cholesterol (HDL-C), apolipoprotein-A (Apo-A), and apolipoprotein-B (Apo-B) were detected by an automatic biochemistry analyzer (Abott, i2000). Peripheral T lymphocyte subsets were tested by flow cytometry (Beckman, FC50), while serum immunoglobulins (IgA, IgG, and IgM) and complements C3 and C4 were estimated on an automatic Specific Proteins Analyzer (Beckman, IMMAGE 800). Samples from healthy stem cell donors were collected as normal controls (NCs).

### ATG-based IST protocol

Enrolled patients were treated with rabbit ATG (r-ATG, Genzyme) and cyclosporine (CsA). An ATG skin test was required by using a slow intravenous infusion of 2.5 mg rATG in 100 mL normal saline (NS) for at least 1 h. rATG was intravenously dosed at 3–4 mg/kg/d from day 1 to day 5. Methylprednisolone (1 mg/kg/d, dosing frequency of q12 h) was administered on day 1 along with rATG, which tapered gradually after 2 weeks if no serum sickness developed. CsA was administered orally at 3–5 mg/kg/d for over 12 months and adjusted in accordance with the target plasma trough concentration (150–250 ng/mL). The dose was tapered 10% every 3 months if the disease was cured and the condition was stable.

### Response criteria

The responses were evaluated every 3 months after IST in line with the international guidelines for AA [2] as complete response (CR), partial response (PR), and nonresponse (NR). The overall response (OR) was defined as CR + PR.

### Definition of events

The definition of the event after IST in this study was as follows: (1) relapse; (2) death; (3) myelodysplastic syndrome (MDS) or leukemia transformation; and (4) switch to HSCT after IST failure.

### Grouping

All the enrolled patients were divided into IST-response (IST-R) and IST-nonresponse (IST-NR) groups based on the efficiency evaluated at 9 months after IST.

### Diet

All patients were given a healthy diet with low salt and fat contents during hospitalization to avoid diet effects on lipid levels.

### Statistical analysis

Statistical analyses were performed using SPSS version 17.0. Categorical data are presented as frequency counts and proportions, and the chi-square test was used to compare the rate of categorical variables. Continuous variables were compared by the Mann–Whitney U test. The cutoff values of Apo-A and HDL-C were calculated by receiver operating characteristic (ROC) curves. The differences in event-free survival (EFS) and overall survival (OS) between subgroups (IST-R and IST-NR, hyper-Apo-A and hypo-Apo-A, hyper-HDL-C and hypo-HDL-C, respectively) were determined by the Kaplan–Meier method with a two-sided log-rank test. The correlation between the IST response and Apo-A, Ig, complements, and T-cell subsets was analyzed using Spearman’s correlation. Logistic regression and Cox regression models were used to analyze multivariate effects on OS and FFS. The absolute value cutoffs of the correlation coefficient (r) were 0.0–0.19, 0.20–0.39, 0.40–0.59, 0.60–0.79, and 0.80–1.0, representing very weak, weak, moderate, strong, and very strong correlations, respectively, as classified by the *British Medical Journal* guidelines [14]. Two-sided *P* < 0.05 was considered significant.

## Results

### Patient characteristics

A total of 27/61 of the newly diagnosed SAA patients were classified as VSAA and enrolled in this study. The cohort, with a median age of 28 (10–57)-years and body mass index (BMI) of 22.2 (17.69–26.95) kg/m^2^, consisted of 29 males. The median duration from diagnosis to treatment was 33 (5–149) days (Table 1). A total of 29 healthy stem cell donors comprised the normal control (NC) group. The median age of the group consisting of 17 males was 31 (11–53)-years-old, and the BMI was 22.6 (17.8–33.6) kg/m^2^. The blood lipid levels of patients with SAA in our cohort were significantly lower than those of NCs; therefore, no pretreatment lipid-lowering therapy was needed. The baseline comparison of patients and NCs is shown in Supplement Table 1.

**Table 1:** Characteristic of patients before ATG-based IST

Characteristic	All (*n* = 61)	IST-R (*n* = 40)	IST-NR (*n* = 21)	Sig (*P* value)
Age (years): median (range)	28(10–57)	27.5(10–55)	30(10–57)	0.313
Gender (male/female)	29/32	20/20	9/12	0.788
BMI (kg/m^2^): median (range)	22.2(17.69–26.95)	21.41(17.69–26.95)	23.54(22.27–26.51)	0.197
SAA/VSAA	34/27	26/14	8/13	***0.041***
Duration (diagnose to IST, days): median (range)	33(5–149)	28.5(5–149)	37(12–139)	0.129
WBC (10^9/L, $\overline{\mathrm X}$ ± s)	1.27 ± 0.59	1.29 ± 0.62	1.25 ± 0.55	0.816
ANC (10^9/L, $\overline{\mathrm X}$ ± s)	0.26 ± 0.15	0.29 ± 0.15	0.20 ± 0.14	***0.033***
RBC (10^12/L, $\overline{\mathrm X}$ ± s)	1.95 ± 0.46	1.93 ± 0.44	2.01 ± 0.49	0.477
HB (g/dL)	59.50 ± 10.87	59.20 ± 10.82	60.05 ± 11.23	0.775
PLT (10^9/L, $\overline{\mathrm X}$ ± s)	7.31 ± 4.16	7.83 ± 4.53	6.33 ± 3.24	0.187
Ret (10^10/L, $\overline{\mathrm X}$ ± s)	1.74 ± 1.38	1.88 ± 1.47	1.48 ± 1.16	0.281
TGs (mmol/L): median (range)	0.83(0.33–5.2)	0.83(0.39–5.2)	0.82(0.33–2.20)	0.744
TC (mmol/L): median (range)	3.51(1.55–6.63)	3.35(1.55–6.63)	3.98(2.53–5.17)	***0.024***
LDL-C (mmol/L): median (range)	1.95(0.73–4.04)	1.81(0.74–4.04)	2.29(0.73–3.12)	***0.043***
HDL-C (mmol/L): median (range)	1.12(0.55–1.82)	0.96(0.55–1.82)	1.23(0.60–1.79)	***0.022***
Apo-A (g/L): median (range)	1.03(0.53–1.60)	0.98(0.53–1.60)	1.21(0.65–1.40)	***0.012***
Apo-B (g/L): median (range)	0.70(0.25–1.39)	0.67(0.26–1.39)	0.83(0.25–1.08)	0.108

### Overall hematological response

Treatment response was assessed 3, 6, and 9 months after ATG-based IST treatment (Table 2). The results showed that the OR at 3 months was 24.6% with 6.6% complete remission (CR) and 18.0% partial remission (PR); the OR at 6 months was 52.5% with 14.8% CR and 37.7% PR; and the OR at 9 months was 65.6% with 23.0% CR and 42.6% PR.

**Table 2:** Response to ATG-based IST at 3, 6, and 9 months

Response (n, %)	3-month (*n* = 61)	6-month (*n* = 61)	9-month (*n* = 61)
Complete response (CR)	4 (6.6)	9 (14.8)	14 (23.0)
Partial response (PR)	11 (18.0)	23 (37.7)	26 (42.6)
Overall response (OR)	15 (24.6)	32 (52.5)	40(65.6)
None response (NR)	46 (75.4)	29 (47.5)	21 (34.4)

### Subgroup baseline characteristics based on 9-month efficiency

All the enrolled patients were divided into IST-response (IST-R) and IST-nonresponse (IST-NR) groups based on the efficiency evaluated at 9 months post-IST: 40 in the IST-R group and 21 in the IST-NR group. The subgroup baseline characteristics based on 9-month efficiency are summarized in Table 1. The age, sex, BMI, duration, white blood cell (WBC), red blood cell (RBC), platelet (PLT), and absolute Ret count were similar across subgroups. However, the ANC and the evaluated severity of AA were remarkably different (all *P* < 0.05). Considering the blood lipid level, TC, LDL-C, HDL-C, and Apo-A were higher in the IST-NR group (all *P* < 0.05).

### Differences in blood lipid levels among groups after ATG-based IST

**Fig. 1:**
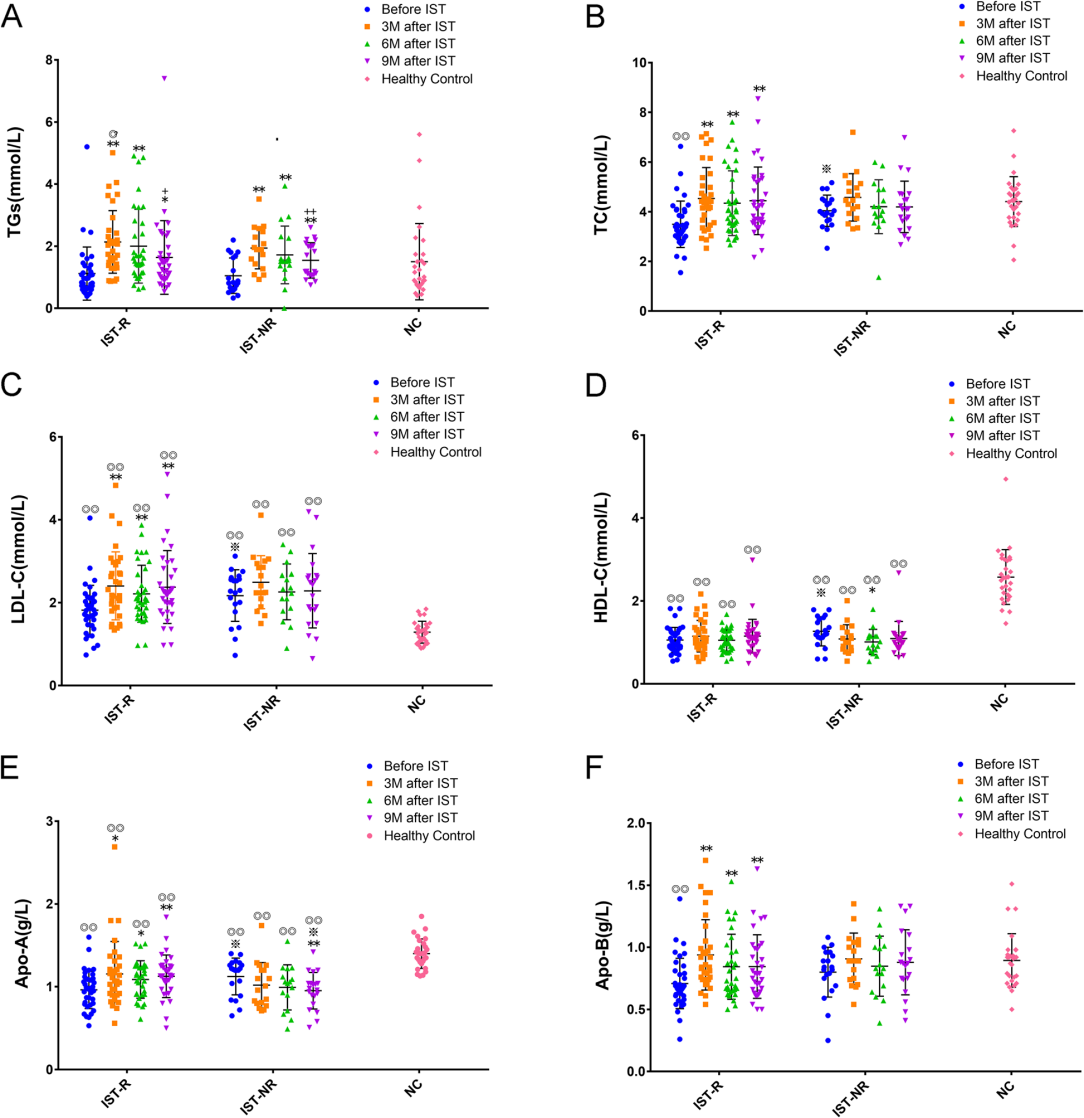
Differences in blood lipids level among groups after ATG-based IST. The changes of peripheral blood lipids, including Triglycerides (TGs, **A**), total cholesterol (TC, **B**), low-density lipoprotein-cholesterol (LDL-C, **C**), high-density lipoprotein-cholesterol (HDL-C, **D**), apolipoprotein A (Apo-A, **E**), and apolipoprotein B (Apo-B, **F** were compared before and 3, 6, and 9 months after ATG-based IST among groups. Samples from healthy donor were tested as normal control group. Compared to before IST, * < 0.05, ** < 0.01; Compared to 3 M after IST, +  < 0.05, +  +  < 0.01; Compared to IST-R, ※ < 0.05; and compared to NC, ◎◎ < 0.01, ◎ < 0.05

The SAA/VSAA patients in our study (both IST-R and IST-NR groups) had a significantly higher LDL-C and lower HDL-C and Apo-A than the healthy donor (NC) pretreatment (*P* < 0.01), and the IST-R group showed a notably lower TC and Apo-B than NC (*P* < 0.01). As stated above, remarkably higher pretreatment serum TC, LDL-C, HDL-C, and Apo-A levels were detected in the IST-NR group than in the IST-R group (all *P* < 0.05). At 3 months after IST, the TGs in both groups increased (*P* < 0.01), which decreased gradually at 6 and 9 months; notable differences were observed 9 months after IST compared to 3 months (all *P* < 0.05) (Fig. 1A). TC and LDL-C increased in the IST-R group at 3, 6, and 9 months after IST compared with pretreatment (*P* < 0.01) but not in the IST-NR group (Fig. 1B, 1C). On the other hand, HDL-C decreased in the IST-NR group and achieved a notable difference 6 months after IST compared with pretreatment (*P* < 0.05), while no changes were observed in the IST-R group (Fig. 1D). Both the Apo-A and Apo-B levels increased in the IST-R group, and significant differences were observed at 3, 6, and 9 months after IST compared with pretreatment (all *P* < 0.05), while Apo-A declined drastically in the IST-NR group, with a significant difference at 9 months after treatment compared to before IST and to that in the IST-R group at 9 months (all *P* < 0.05). Strikingly, no obvious changes were noted in Apo-B expression after treatment in the IST-NR group (Fig. 1E and F).

### Altered serum IgA, IgG, IgM, C3, and C4 levels after ATG-based IST

**Fig. 2:**
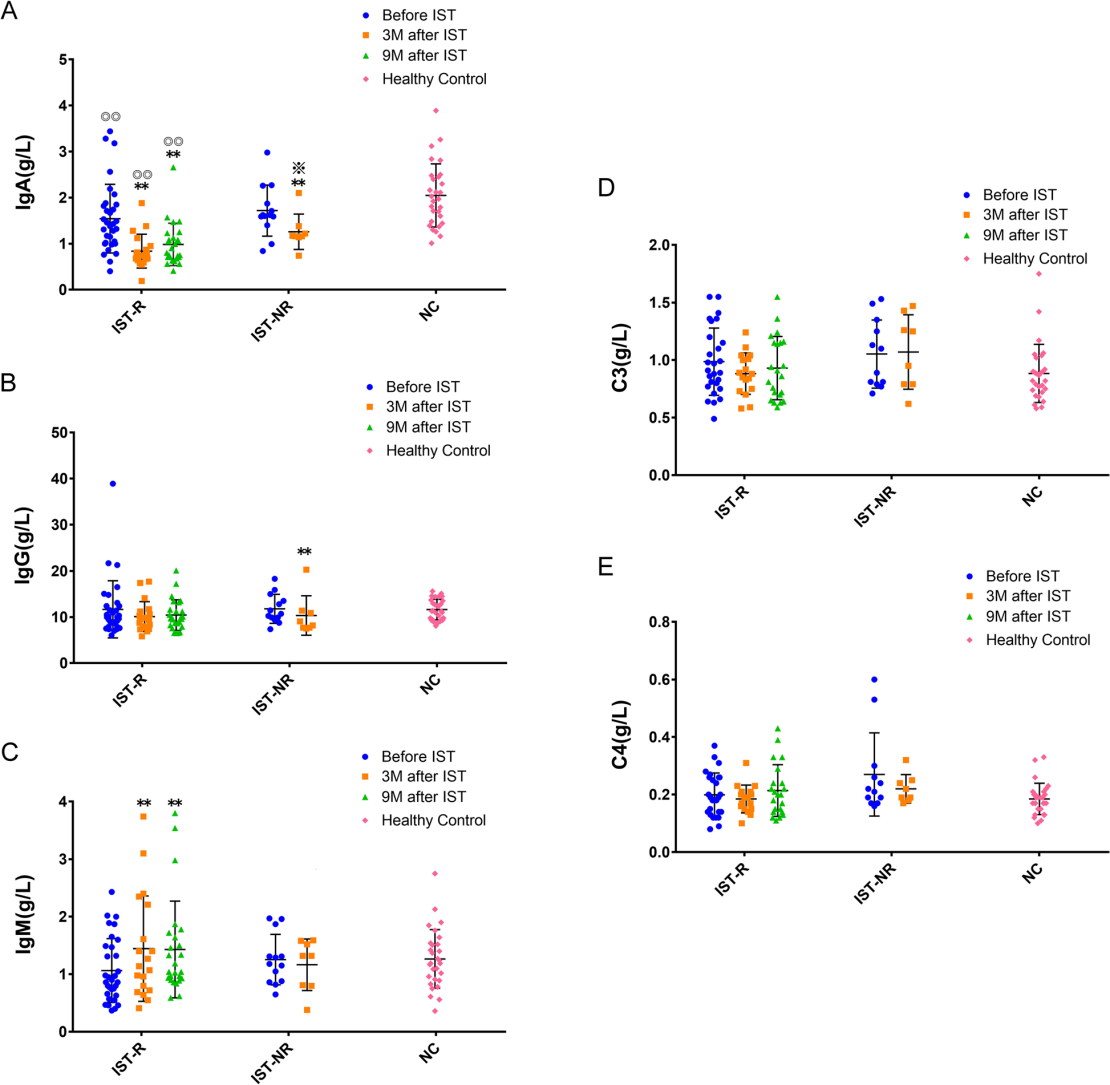
The changes of serum IgA (**A**), IgG (**B**), IgM (**C**), C3 (**D**), and C4 (**E**) were compared among groups based on ATG-based IST. Compared to before IST, ** < 0.01; Compared to IST-R, ※ < 0.05; Compared to NC, ◎◎ < 0.01, ◎ < 0.05

Limited data are available on immunoglobulin and complement levels after reexamination at 6 and 9 months in the IST-NR group. Herein, we compared the differences between groups based on 3-month data. It was found that the IST-R group had a markedly decreased IgA population compared to the NC group, and there were no differences between the IST-R and IST-NR groups on these indexes before the treatment. At 3 months after IST, both groups showed decreased IgA (*P* < 0.01), but the level in the IST-R group was much lower (*P* < 0.05) (Fig. 2A). In addition, the IST-NR group showed decreased expression of IgG after 3 months (*P* < 0.01) (Fig. 2B), while the IST-R group had an increased level of IgM (*P* < 0.01) (Fig. 2C). Additionally, no obvious changes were detected in complement C3 and C4 after treatment, and no difference was found between the groups (Fig. 2D and E).

### Changes in peripheral T lymphocytes after ATG-based IST

**Fig. 3:**
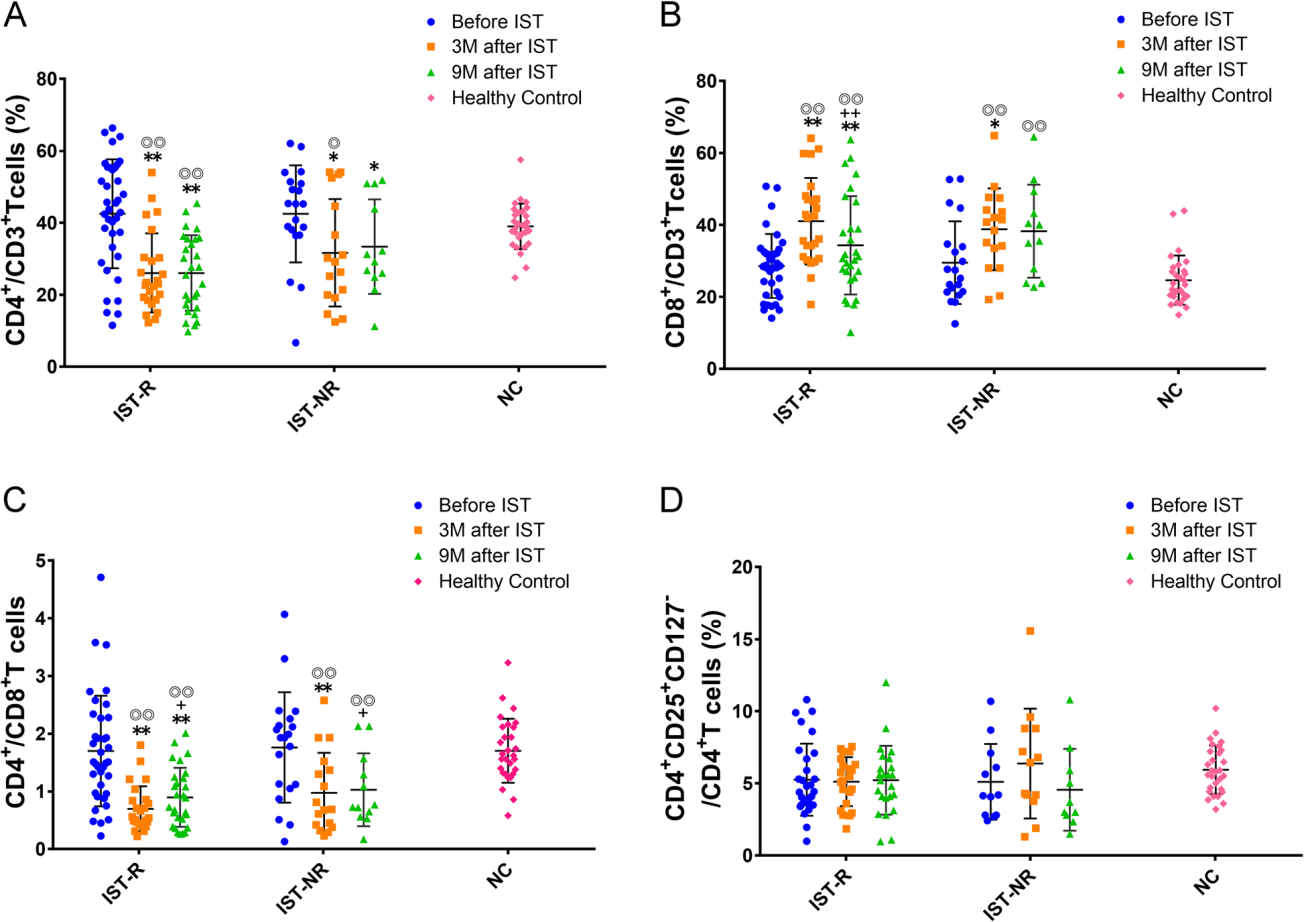
Changes in peripheral T lymphocytes after ATG-based IST Changes of peripheral CD4 + CD3 + helper T cells (**A**), CD8 + CD3 + suppressor T cells (**B**), ratio of CD4 + /CD8 + (**C**), and CD4 + CD25 + CD127- regulatory T cells (**D**) were compared among groups after ATG-based IST. Compared to before IST, * < 0.05, ** < 0.01; compared to 3 M after IST, +  < 0.05; compared to NC, ◎◎ < 0.01, ◎ < 0.05

Although a higher CD8^+^CD3^+^ T-cell number was estimated in the IST-R group (*P* = 0.053) than NC before treatment, no significant differences were found in the pretreatment level of CD4^+^CD3^+^, CD8^+^CD3^+^ T lymphocytes, the ratio of CD4^+^/CD8^+^, and CD4^+^CD25^+^CD127^−^ regulatory T cells. These levels also differed between the IST-R and IST-NR groups. After ATG-based IST treatment, the CD4^+^CD3^+^ helper T cells decreased in both the IST-R and IST-NR groups (*P* < 0.01 or *P* < 0.05), but no differences were noted between the groups at 3 and 9 months after IST (Fig. 3A). Surprisingly, the ratio of CD8^+^CD3^+^ suppressor T cells increased 3 months after IST in both groups (*P* < 0.01 or *P* < 0.05). However, in the IST-R group, the percentage decreased 9 months after IST (*P* < 0.01) but was still higher than pretreatment (*P* < 0.01); in the IST-NR groups, the percentage was retained at a high level 9 months after IST compared to 3 months and before IST (Fig. 3B). Next, the ratio of CD4^+^/CD8^+^ cells decreased 3 months after therapy in both groups (*P* < 0.01) but increased gradually 9 months after IST compared to the 3-month data (*P* < 0.05) (Fig. 3C). Furthermore, no significant changes were observed in CD4^+^CD25^+^CD127^−^ regulatory T cells between the groups at any time point (Fig. 3D).

### Predicting the 9-month IST response based on Apo-A and IgA levels before and 3 months after the treatment

To predict the 9-month response early, a correlation analysis between IST response (9 months) and Apo-A, HDL-C, Ig, complements, and T-cell subsets pre- and 3 months post-treatment was conducted. The results showed that the 9-month IST response had a moderate negative correlation with IgA levels at 3 months (Spearman’s r = -0.516, *P* = 0.006) and a negative correlation with pretreatment Apo-A levels (Spearman’s r = -0.380, *P* = 0.004). In addition, HDL-C pretreatment showed a weak negative correlation with the 9-month IST response (Spearman’s r = -0.297, *P* = 0.025), as for the other parameters, Ig, complements, and T-cell subsets, did not show any correlations.

### OS and EFS differences based on the 9-month IST response

**Fig. 4:**
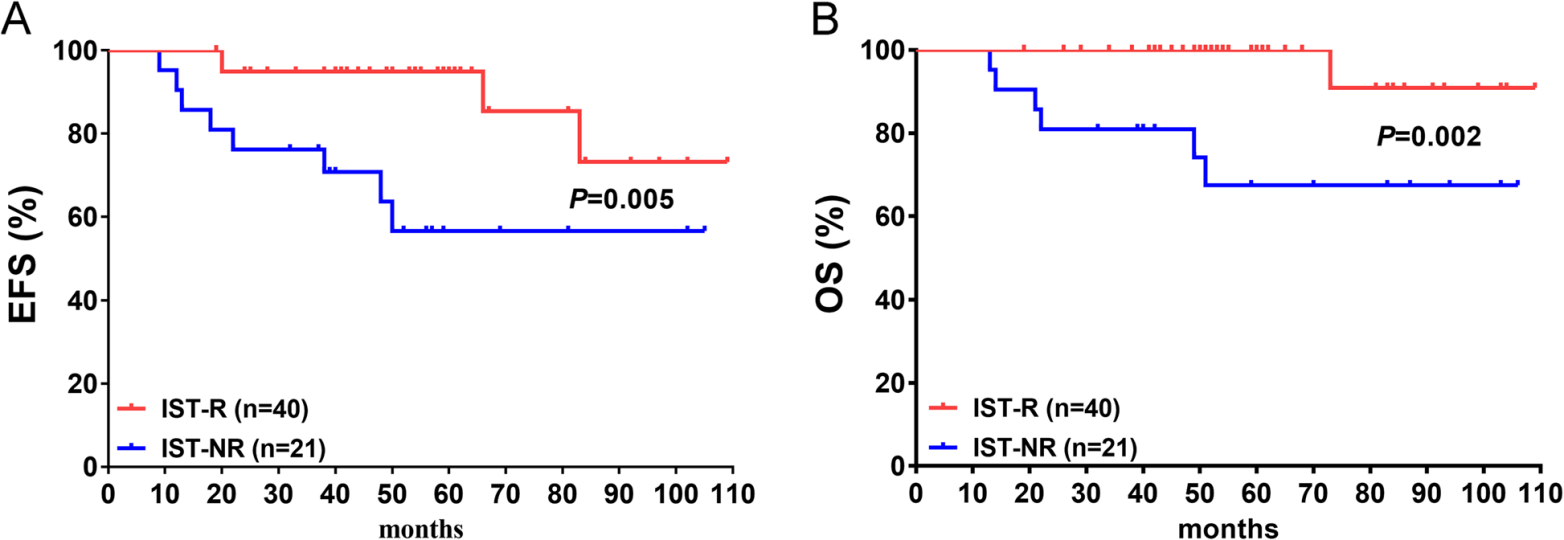
Overall survival (OS) and event free survival (EFS) difference based on 9-month IST response. There was a significantly differences on EFS (*P* = 0.005, **A**) and OS (*P* = 0.002, **B**) between IST-R and IST-NR groups. The estimated 8.5-year EFS was 73.2% and 56.6% in IST-R and IST-NR group, respectively; and the estimated 8.5-year OS was 90.9% and 67.5%, respectively

The median follow-up of this study was 55 (13–109) months, and 4 and 8 events were observed in the IST-R (*n* = 40) and IST-NR (*n* = 21) groups, respectively (Supplement Table 2). A significant difference was noted in the events between the IST-R and IST-NR groups (all *P* < 0.05), but no difference was noted in death. Additionally, a significant difference was obtained between EFS and OS based on the IST response (*P* = 0.005 and *P* = 0.002, respectively) (Fig. 4A and B). The estimated 8.5-year EFS was 73.2% and 56.6% in the IST-R and IST-NR groups, respectively, and the estimated 8.5-year OS was 90.9% and 67.5%, respectively.

### OS and EFS difference based on serum Apo-A level

**Fig. 5:**
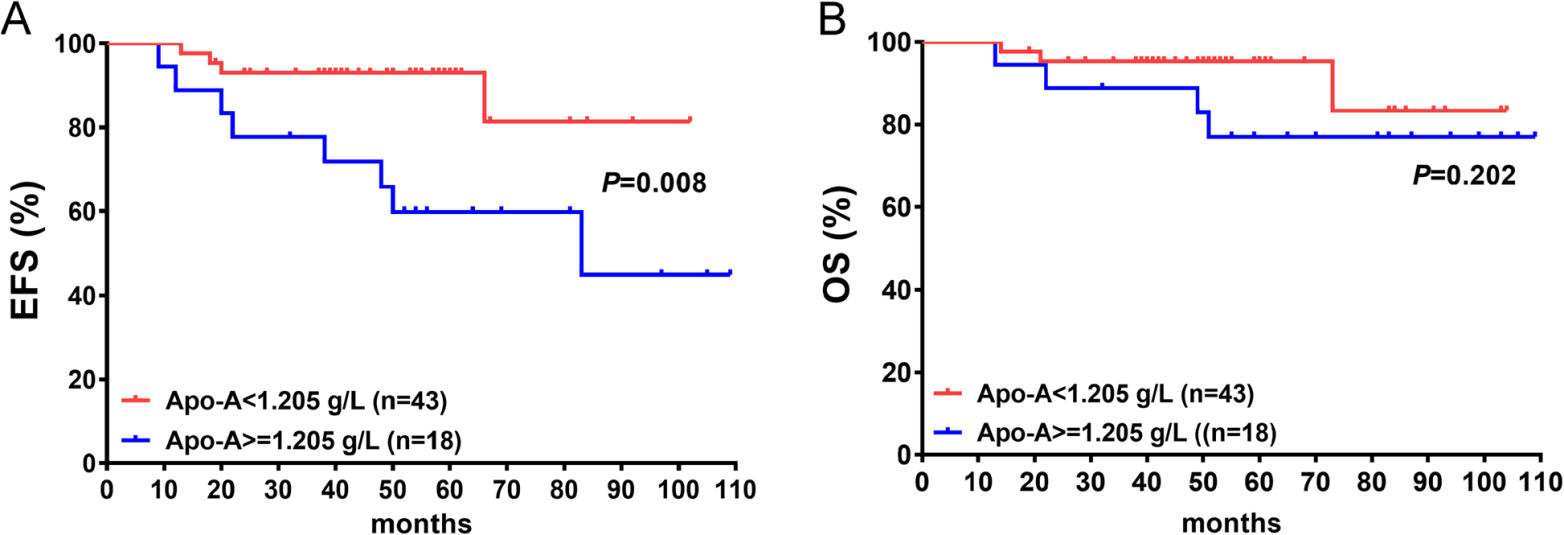
Overall survival (OS) and event free survival (EFS) based on serum Apo-A level. There was a significantly differences on EFS (*P* = 0.008, **A**) based on serum Apo-A level, but not OS (*P* = 0.202, **B**). The estimated 8.5-year EFS was 81.3% in group with Apo-A < 1.205 g/L and 44.9% in group with Apo-A ≥ 1.205 g/L; and the estimated 8.5-year OS was 83.4% and 77.0% in group with Apo-A < 1.205 g/L or ≥ 1.205 g/L, respectively

Considering the dramatic changes between the IST-R and IST-NR groups, a ROC curve defined a cutoff value of 1.205 g/L that exhibited 60% sensitivity and 83% specificity in predicting EFS with an area under the curve (AUC) of 0.715 (*P* = 0.034) before treatment. Finally, 1.205 was selected rather than Youden’s index as the cutoff value for balanced subgrouping. Subsequently, all the enrolled patients were classified into two subgroups based on the level of Apo-A before treatment: hyper-Apo-A (the level of Apo-A ≥ 1.205 g/L) and hypo-Apo-A (the level of Apo-A < 1.205 g/L). Herein, the baseline characteristics based on Apo-A levels were compared, and the only difference was attributed to age. The patients with a younger median age had a higher serum Apo-A (Table 3). Surprisingly, the patients with Apo-A ≥ 1.205 g/L had a poorer EFS than those with Apo-A < 1.205 g/L (*P* = 0.008) (Fig. 5A), but no differences were observed in OS (Fig. 5B). The estimated 8.5-year EFS was 81.3% in the group with Apo-A < 1.205 g/L and 44.9% in the group with Apo-A ≥ 1.205 g/L, and the estimated 8.5-year OS was 83.4% and 77.0% in the group with Apo-A < 1.205 g/L or ≥ 1.205 g/L, respectively.

**Table 3:** Baseline characteristic of patients before IST based on serum Apo-A level

Characteristic	All (n = 61)	Hyper-Apo-A (*n* = 18)	Hypo-Apo-A (*n* = 43)	Sig (*P* value)
Age (years): median (range)	28.0(10–57)	21.5(10–56)	29.0(13–57)	***0.015***
Gender (male/female)	29/32	8/10	21/22	0.786
BMI (weight/hight^2): median (range)	22.2(17.69–26.95)	22.27(22.27–24.80)	21.41(17.69–26.96)	0.517
SAA/VSAA	40/21	11/7	29/14	0.769
Duration (diagnose to IST, days): median (range)	33.0(5–149)	34.5(13–98)	27.0(5–149)	0.452
WBC (10^9/L, $\overline{\mathrm X}$ ± s)	1.27 ± 0.59	1.20 ± 0.42	1.31 ± 0.65	0.769
ANC (10^9/L, $\overline{\mathrm X}$ ± s)	0.26 ± 0.15	0.24 ± 0.17	0.27 ± 0.14	0.620
RBC (10^12/L, $\overline{\mathrm X}$ ± s)	1.95 ± 0.46	2.06 ± 0.36	1.91 ± 0.49	0.116
HB (g/dL)	59.50 ± 10.87	60.89 ± 10.86	58.91 ± 10.95	0.261
PLT (10^9/L, $\overline{\mathrm X}$ ± s)	7.31 ± 4.16	6.39 ± 3.50	7.70 ± 4.40	0.396
Ret (10^10/L, $\overline{\mathrm X}$ ± s)	1.74 ± 1.38	1.55 ± 1.13	1.82 ± 1.47	0.486

### EFS and OS risk evaluation based on patients’ baseline characteristics

A logistic regression model was applied to distinguish the putative elements affecting EFS or OS. Considering that Apo-A is a component of HDL-C and that the changes in Apo-A between the IST-R and IST-NR groups are distinct, HDL-C was ruled out to avoid its interference with Apo-A during logistic regression. First, all putative factors, such as sex, age, severity of disease, duration from diagnosis to IST, level of WBC, ANC, RBC, HB, PLT and Ret before treatment, and Apo-A subgroup based on 1.205 were entered into logistic regression separately, and then variables with *P* value < 0.1 were added to the logistic regression (Table 4). Subsequently, logistic regression identified Apo-A ≥ 1.205 as the only predictor associated with poor EFS [odds ratio (OR) = 7.800, 95% confidence interval (CI): 1.949–31.216, *P* = 0.004]. The same results were obtained by the Cox regression model [hazard ratio (HR) = 4.445, 95% CI: 1.329–14.837, *P* = 0.015]. None of the factors were associated with OS (Table 5).

**Table 4:** Factors association with EFS (logistic regression separately)

	Odds Ratio	95% Confidence Limits		*P*-value
Male vs Female	0.885	0.250	3.128	0.847
Age (years old)	0.957	0.905	1.012	*0.125*
SAA vs VSAA	0.941	0.247	3.582	0.929
Duration from diagnose to IST (months)	1.002	0.982	1.022	*0.856*
Apo-A < 1.205 vs Apo-A ≥ 1.205 (g/L)	7.800	1.949	31.216	***0.004***
WBC level (10^9/L)	2.018	0.716	5.693	*0.184*
ANC level (10^9/L)	1.079	0.016	74.554	0.972
RBC level (10^12/L)	0.579	0.129	2.601	0.475
HB level (g/dL)	0.984	0.928	1.044	0.594
PLT level (10^e9/L)	0.867	0.714	1.053	0.151
Ret level (10^e10/L)	1.021	0.646	1.614	0.929

**Table 5:** Factors association with OS (logistic regression separately)

	Odds Ratio	95% Confidence Limits		*P*-value
Male vs Female	0.320	0.057	1.797	*0.196*
Age (years old)	0.967	0.903	1.034	*0.326*
SAA vs VSAA	0.283	0.032	2.526	*0.259*
Duration from diagnose to IST (months)	0.998	0.973	1.025	*0.909*
Apo-A < 1.205 vs Apo-A ≥ 1.205 (g/L)	3.810	0.757	19.172	*0.105*
WBC level (10^9/L)	2.952	0.829	10.516	***0.095***
ANC level (10^9/L)	64.280	0.160	25,881.145	*0.174*
RBC level (10^12/L)	1.154	0.209	6.368	*0.869*
HB level (g/dL)	1.016	0.944	1.094	*0.667*
PLT level (10^e9/L)	0.948	0.768	1.169	*0.616*
Ret level (10^e10/L)	1.178	0.692	2.003	*0.546*

### OS and EFS difference based on serum IgA level

**Fig. 6:**
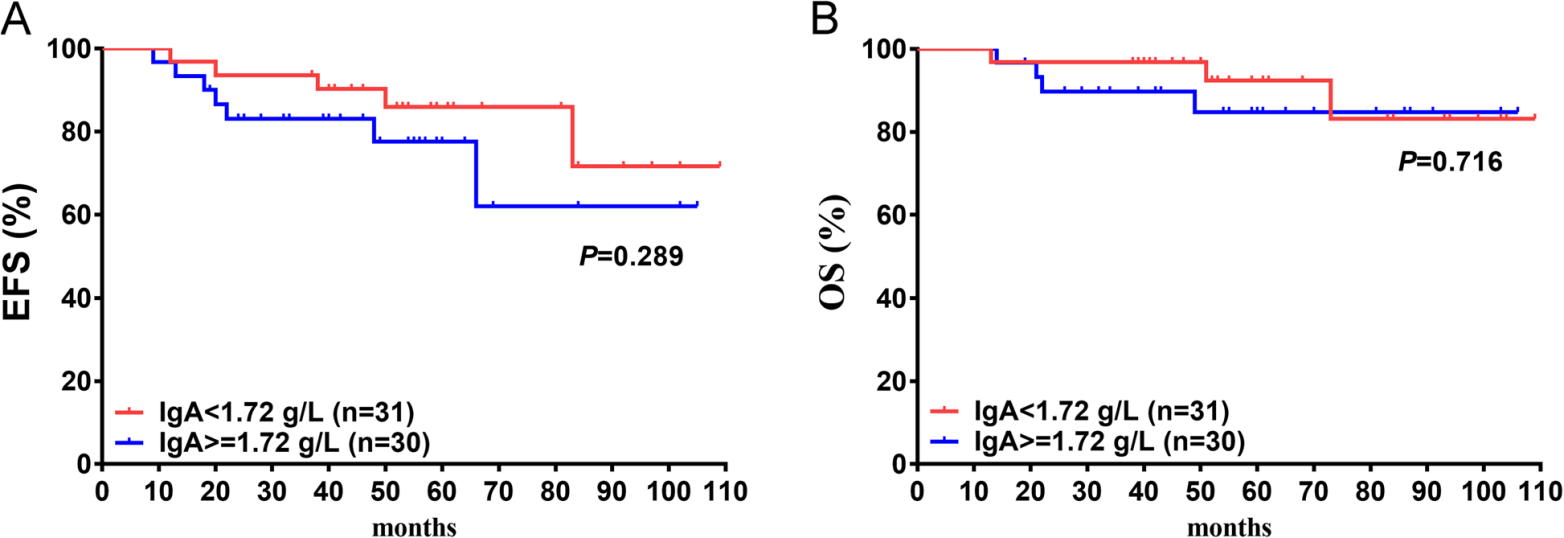
Overall survival (OS) and event free survival (EFS) based on serum IgA level. There was no significantly differences on EFS nor OS based on serum IgA level. The estimated 8.5-year EFS was 71.6% in group with IgA < 1.72 g/L and 62.0% in group with IgA ≥ 1.72 g/L; and the estimated 8.5-year OS was 83.1% and 84.8% in group with IgA < 1.72 g/L or ≥ 1.72 g/L,respectively

The changes in IgA in the IST-R groups did not differ significantly compared to those in the IST-NR group. Thus, we used an average of IgA before treatment as the cutoff value based on the ROC with no discriminating ability to predict the EFS (AUC = 0.418, *P* = 0.551). The patients with IgA levels ≥ 1.72 g/L showed poorer EFS than those with IgA < 1.72 g/L, although no statistically significant difference was noted (*P* = 0.289, Fig. 6A); a similar observation was made with OS (*P* = 0.716, Fig. 6B).The estimated 8.5-year EFS was 71.6% in the group with IgA < 1.72 g/L and 62.0% in the group with IgA ≥ 1.72 g/L, and the estimated 8.5-year OS was 83.1% and 84.8% in the group with IgA < 1.72 g/L or ≥ 1.72 g/L, respectively.

## Discussion

Reportedly, the hematopoietic activity within the bone marrow in children with AA was correlated with the pretreatment serum lipid level, and TC < 150 mg/dL indicated a poor response to IST drugs [15]. In addition, the lipid peroxidation index (malondialdehyde, MAD) was higher in children with AA, in contrast to the lower antioxidant (glutathione, GSH) compared to NC [16]. Nonetheless, the characteristics of lipid changes during ATG-based IST in patients with severe or very severe grades and their significance have yet to be elucidated. In our cohort, the majority were adult patients (54/61, 88.5% aged > 16 years), with relatively lower TG and TC levels before treatment compared to the healthy controls. In addition, a significantly lower TC was detected in patients’ response to IST, which was inconsistent with the literature [17]. Feng et al. found that an increased frequency of HSC, monocytes and granulocytes was observed in the peripheral blood in LDL receptor knockout (LDLr^−/−^) mice with higher cholesterol levels, as well as a higher number of CD34^+^ stem cells mobilized in hypercholesterolemia patients than in normal cholesterol patients [18, 19], indicating that hypercholesterolemia and hyper-LDL exhibited protective effects on HSC proliferation and differentiation. These findings were consistent with R patients with increased levels of TC and LDL-C after ATG-based IST in our cohort, while no changes in TC or LDL-C were detected in NR patients before and after treatment. The Apo-A level increased in R patients but was opposite in the NR group, indicating a correlation between Apo-A and the varied responses. In this study, we selected 1.205 g/L as the cutoff value for Apo-A and found that patients with lower Apo-A pretreatment levels achieved a better EFS but no difference in OS. One possible explanation is that patients with upregulated Apo-A may benefit from the accelerated clearance of increased blood lipids and protect hematopoiesis from the toxicity of free radical species released during lipid metabolism [20].

However, why the blood lipid level increases after ATG-based IST has yet to be elucidated. CsA, a specific inhibitor of calcineurin, was shown to decrease glucose uptake in human subcutaneous adipocytes [21] and promote glycogen synthesis [22, 23], which might further accelerate lipolysis in adipocytes [24]. Thus, it is speculated that CsA influences lipid metabolism in renal transplant recipients, and secondary hypercholesterolemia and hypertriglyceridemia can be corrected with the application of simvastatin [25]. For SAA/VSAA containing abundant adipocytes in the bone marrow, the change in marrow adipocyte lipolysis should also be investigated. In addition, CsA was confirmed to be associated with the concentration of Apo-A [26], suggesting that in the IST-R group, the effectiveness of CsA could be reflected by the adjusted lipid metabolism, especially the level of Apo-A. In addition to its role in cholesterol trafficking, Apo-A, a multifunctional protein, participates in inflammatory and immune responses [27]. It also stimulates the proliferation of monocytes, granulocytes and their progenitor cells and bone marrow MSCs [28] and supports MSC survival under stress conditions [29], which might be beneficial for hematopoiesis recovery [20]. Some studies revealed that exogenous-free Apo-A alters the characteristics of progenitor cell populations [30]. In addition, LDLr^−/−^Apo-A I^−/−^mice fed an atherogenic diet manifested increased T lymphocyte cell proliferation and activation compared to LDLr^−/−^ mice, which can be reversed by treatment with helper-dependent adenovirus expressing Apo-A I [27]. Therefore, we speculated that patients with increased Apo-A levels may achieve a response by affecting T lymphocyte populations.

Furthermore, AA is believed to be a cytotoxic T-cell-mediated bone marrow failure disorder [31], and the serum IgG level was lower in AA [32]. To date, a few studies have investigated the changes in T-cell subsets and serum immunoglobulin levels other than IgG in SAA/VSAA after ATG-based IST. In the present study, we did not observe any significant increase in CD8^+^ T-cell number (% of CD3^+^ T cells) before treatment in SAA/VSAA patients, but a decrease in CD4^+^ T cells (% of CD3^+^) and an increase in CD8^+^ T-cell number (% of CD3^+^) were noted 3 months after IST, which may be ascribed to the selective clearance effect of ATG on CD4^+^ T cells [33]. The role of IgA in the pathological process of AA has not yet been clarified. Reportedly, IgA1 regulates the expression of its receptors and triggers monocyte-derived dendritic cell maturation, followed by the activation of cytotoxic CD8^+^ T cells [34], while the quantity and function of myeloid dendritic cells were confirmed to be abnormally expressed in SAA [35, 36]. In this study, we considered that the low expression of IgA in SAA/VSAA may exert a protective effect, and the decreased IgA level in the IST-R patients may be beneficial to the decreased CD8^+^ population at 9 months after treatment. The additional correlation analysis also indicated that the therapy was a moderate correlation between the 3-month IgA level and the 9-month response.

### Comparisons with other studies and what does the current work add to the existing knowledge

Although lipid metabolism has received extensive attention in recent years, there are few published studies about lipid changes in AA [15, 37]. Our study is the first to describe the changes in peripheral lipids in SAA patients who received IST with r-ATG and CsA and to evaluate Apo-A as a prognostic marker. In addition, we discovered a negative correlation between the early IgA level and IST efficacy.

### Study strength and limitations

We defined Apo-A as a potential prognostic biomarker for SAA patients who received ATG-based IST therapy through serum lipid changes, which may open up a novel path to enhance the current efficacy of ATG-based IST by modulating lipid metabolism. The main deficiency originated from the insufficient sample size, particularly in subgroup analyses. In addition, we further investigated the correlation between marrow adipocytes and peripheral lipids by detecting substances secreted into the blood by adipocytes, such as leptin.

## Conclusion

In conclusion, the current study found that serum Apo-A is a potential prognostic biomarker for newly diagnosed < 60-year-old SAA/VSAA patients who received ATG-based IST, and the level of 3-month IgA can also be an indicator for the 9-month IST response. Lipid-lowering therapy prior to ATG-based IST may improve the prognosis of patients with SAA.

## Supplementary Information

**Additional file 1:** **Supplement Table 1.** Baseline comparison of patients and healthy donors.

**Additional file 2: Supplement Table 2. **Events happened in IST-R and IST-NR group.

## Data Availability

Data and materials in this manuscript are available from the corresponding author upon reasonable request.
