# Supplementary material for: Apolipoprotein-A is a potential prognostic biomarker for severe aplastic anemia patients treated with ATG-based immunosuppressive therapy: a single-center retrospective study
Source: Lipids Health Dis. 2022 Oct 4;21:93. doi: 10.1186/s12944-022-01703-0 (PMC9531379; doi:10.1186/s12944-022-01703-0)
Supplement: Supplementary file 1 — Additional file 1: Supplement Table 1. Baseline comparison of patients and healthy donors. [file 12944_2022_1703_MOESM1_ESM.docx]

**Supplement Table 1: Baseline comparison of patients and healthy donors**

| Characteristic | Patients (n=61) | NC (n=29) | Sig (*P* value) |
| --- | --- | --- | --- |
| Age (years): median (range) | 28(10-57) | 31 (11–53) | *0.800* |
| Gender (male/female) | 29/32 | 17/14 | *0.225* |
| BMI (kg/m^2^) : median (range) | 22.2(17.69-26.95) | 22.6 (17.8–33.6) | *0.679* |
| WBC (10^9/L, ‾x±s) | 1.27±0.59 | 5.95±1.36 | *0.000* |
| ANC (10^9/L, ‾x±s) | 0.26±0.15 | 3.47±0.99 | *0.000* |
| RBC (10^12/L, ‾x±s) | 1.95±0.46 | 4.83±0.54 | *0.000* |
| HB (g/dL, ‾x±s) | 59.50±10.87 | 143.72±19.64 | *0.000* |
| PLT (10^9/L, ‾x±s) | 7.31±4.16 | 225±52.55 | *0.000* |
| Ret (10^12/L, ‾x±s) | 1.74±1.38 | 6.23±2.78 | *0.000* |
| TGs (mmol/L): median (range) | 0.83(0.33-5.2) | 1.01(0.41-5.6) | *0.111* |
| TC (mmol/L): median (range) | 3.51(1.55-6.63) | 4.45(2.06-7.26) | *0.001* |
| LDL-C (mmol/L): median (range) | 1.95(0.73-4.04) | 1.28(0.90-1.85) | *0.000* |
| HDL-C (mmol/L): median (range) | 1.12(0.55-1.82) | 2.56(1.46-4.94) | *0.000* |
| Apo-A (g/L): median (range) | 1.03(0.53-1.60) | 1.40(1.13-1.85) | *0.000* |
| Apo-B (g/L): median (range) | 0.70(0.25-1.39) | 0.92(0.50-1.51) | *0.002* |

Note：healthy stem cell donors are comprised as the normal control (NC) group
