# Supplementary material for: Apolipoprotein-A is a potential prognostic biomarker for severe aplastic anemia patients treated with ATG-based immunosuppressive therapy: a single-center retrospective study
Source: Lipids Health Dis. 2022 Oct 4;21:93. doi: 10.1186/s12944-022-01703-0 (PMC9531379; doi:10.1186/s12944-022-01703-0)
Supplement: Supplementary file 2 — Additional file 2: Supplement Table 2. Events happened in IST-R and IST-NR group. [file 12944_2022_1703_MOESM2_ESM.docx]

**Supplement Table2. Events happened in IST-R and IST-NR group**

| Event | IST-R (n=40) | IST-NR (n=21) | sig |
| --- | --- | --- | --- |
| Relapse | 3 | 2 | χ2=0.075, *P*=0.784 |
| Death | 1 | 6 | χ2=9.214, *P*=0.005 |
| Total Events | 4 | 8 | χ2=6.879, *P*=0.016 |
